# Reoperative arch-first total arch repair after previous acute type A aortic dissection repair

**DOI:** 10.1016/j.xjtc.2025.03.015

**Published:** 2025-03-28

**Authors:** Suguru Ohira, Ramin Malekan, Masashi Kai, Sooyun Caroline Tavolacci, Vasiliki Gregory, Junichi Shimamura, Igor Laskowski, Steven L. Lansman, David Spielvogel

**Affiliations:** a Division of Cardiothoracic Surgery, Department of Surgery, Westchester Medical Center, New York Medical College, Valhalla, NY; b New York Medical College, Valhalla, NY; c Division of Cardiac Surgery, Beth Israel Deaconess Medical Center, Boston, Mass; d Division of Vascular Surgery, Department of Surgery, Westchester Medical Center, New York Medical College, Valhalla, NY

**Keywords:** aortic arch, aortic dissection, reoperation

## Abstract

**Objective:**

We sought to review the outcomes of our arch-first total aortic arch repair (TAR) using a trifurcated graft after previous acute type A aortic dissection (ATAD) repair.

**Methods:**

From February 2006 to June 2024, 62 patients underwent reoperative TAR after ATAD repair. The first-stage TAR includes axillary artery cannulation, minimal dissection without aortic crossclamping, myocardial protection using systemic potassium and retrograde blood cardioplegia, an arch-first technique with deep hypothermia (20 °C), and construction of a classical elephant trunk through a partial transverse incision distally or proximally to old distal aortic anastomosis.

**Results:**

The median age at reoperative TAR was 63.5 years. The median interval from initial ATAD repair to reoperative TAR was 3 years. A concomitant procedure was performed in 20 patients (32.3%). The median cardiopulmonary bypass and lower body circulatory arrest times were 227.5 and 97 minutes, respectively. Operative mortality was 1.6% (n = 1/62), as was the incidence of stroke (1.6%) and renal-replacement therapy (3.2%). Stage II repair was performed or planned in 49 patients (open repair [above the celiac axis in most patients], n = 42; endovascular, n = 3; endovascular converted to open repair, n = 2; and waiting for repair, n = 2). Median interval between staged procedures was 63 days [interquartile range, 36, 134]. Mortality of stage II procedure was 4.3% (n = 2/47) with no spinal cord injury. Kaplan-Meier analysis showed that estimated survival at 5 years was 82.7 ± 6.7%.

**Conclusions:**

Our reoperative TAR is safe in the setting of residual dissection that minimizes dissection of the cardiac structures, simplifies the distal anastomosis, and protects vital organs.

**Figure:**
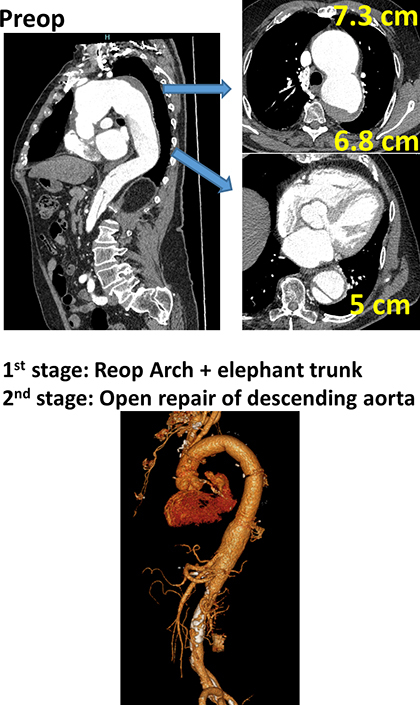
Reoperative arch repair followed by open extent I thoracoabdominal aortic repair.

**Central Message**

Reoperative arch-first total arch repair for previous type A dissection repair minimizes dissection of the great vessels, simplifies the distal anastomosis, and protects vital organs.

**Perspective**

Reoperative arch-first total arch repair using a trifurcated graft enables in situ repair while a bifurcated graft technique allows a shorter circulatory arrest time of the lower body, which might be beneficial in patients who need concomitant complex proximal or arch repair. Insertion of classical elephant trunk followed by staged distal intervention showed favorable follow-up outcomes.

Surgical outcomes of acute type A aortic dissection have been improving as the result of advances in diagnosis and treatment over the past decades.^1^^,^^2^ Most centers perform either ascending or hemiarch repair in order to balance the safety and efficacy of intervention as emergent acute type A aortic dissection (ATAD) repair is a life-saving operation.^1^, ^2^, ^3^, ^4^ With regard to follow-up after initial ATAD repair, a downstream aorta with residual dissection can dilate, with an incidence ranging from 2.7% to 19.5% of patients, mainly as a result of the extension of initial dissections beyond the aortic arch to the distal aorta or new entry from the distal anastomosis.^3^, ^4^, ^5^, ^6^, ^7^, ^8^, ^9^, ^10^, ^11^

Although open repair is considered as a gold standard for chronic aortic dissection as a definitive repair, there has been the perception that reoperative surgery for total aortic arch repair (TAR) carries unacceptable risks of mortality and major morbidity, which has underpinned alternative approaches, such as debranching hybrid and total endovascular procedures.^12^, ^13^, ^14^ Advent of endovascular procedures provides more options in patients with residual dissection; however, the specific complications, such as endoleaks, stent-induced new entry, stent migration or collapse, or occlusion of aortic branches, can potentially make aortic pathology more complex.^12^, ^13^, ^14^, ^15^, ^16^ In addition, long-term surveillance is necessary as durability of endovascular repair is still not fully understood in chronic aortic dissection.^14^^,^^16^ Recent studies from aortic centers showed the safety of reoperative TAR for any pathology where operative mortality ranges from 2.0 to 8.3%.^17^, ^18^, ^19^, ^20^, ^21^ Because there is an increasing volume of patients referred for reinterventions on downstream aorta after previous ATAD repair, we sought to focus on the strategies and outcomes of reoperative TAR for residual dissection.

## Methods

This study is a retrospective analysis and was approved by the New York Medical College Internal Review Board, with a waiver of individual consent (#14209; approval date May 2, 2020). From February 2006 to June 2024, 62 patients had reoperative TAR for residual dissection after ATAD repair. The 2-staged procedures were standard to complete aortic repair. The single surgical team (5 surgeons) almost equally performed these procedures with the same reproducible method shared across the team. Indications for arch repair were presence of aortic diameter ≥55 mm in the aortic arch, and typically ≥60 mm in the descending aorta.

### Surgical Techniques

#### Reoperative TAR

**Figure 1:**
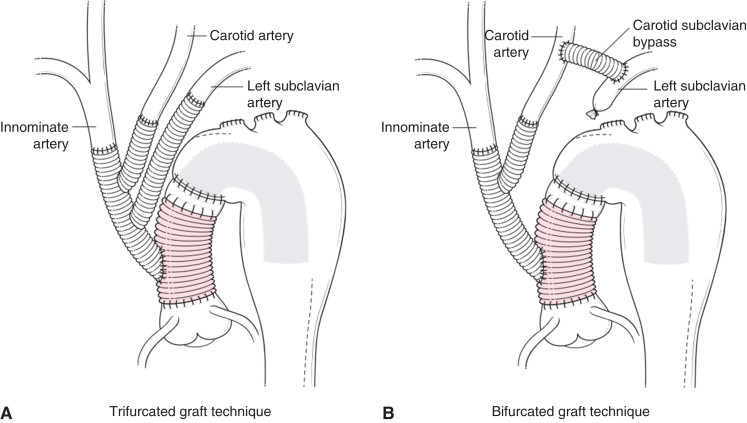
Reoperative aortic arch repair using (A) a trifurcated graft (12 × 8 × 8 mm) and (B) bifurcated graft technique (12 × 8 mm). In these figures, incision on the aorta was made distal to the old distal anastomosis (Technique II). An ascending graft in *red* shows a previous graft. An elephant trunk is inserted to the descending aorta with or without fenestration.

**Figure 2:**
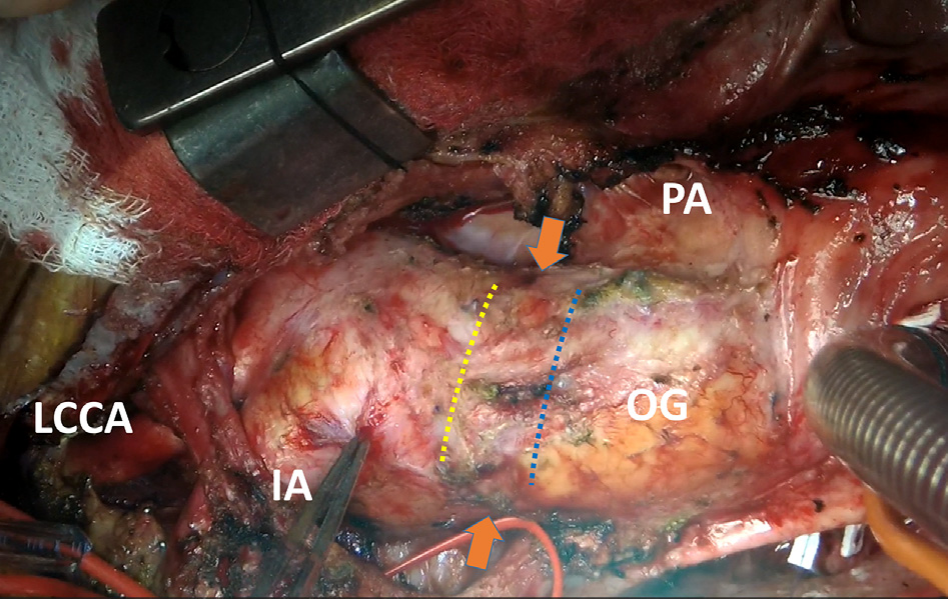
Intraoperative picture. *Orange arrows* show a previous distal aortic anastomosis. A partial transverse incision can be made either proximally in the old graft (Technique I, *blue dots*) or in the aorta just distal to the old graft (Technique II, *yellow dots*), depending on the length of ascending graft. *LCCA*, Left common carotid artery; *IA*, innominate artery; *OG*, old ascending graft; *PA*, pulmonary artery.

Right axillary artery was directly cannulated. Minimal dissection of the mediastinal structures was performed because aortic crossclamping is not necessary for isolated arch repair. After reaching deep hypothermia (bladder temperature, 18-20 °C), systemic potassium was given (40-60 mEq) and circulatory arrest of the lower body was initiated. Unilateral antegrade cerebral perfusion (ACP) was established via right axillary cannulation. Retrograde blood cardioplegia was given through the period of myocardial ischemia. A 12- × 8- × 8-mm trifurcated graft (Hemashield: Getinge) was used to reconstruct all head vessels (trifurcated group) individually or was trimmed to make a bifurcated graft to revascularize first and second arch vessels (bifurcated group) (Figure 1). After its completion, bilateral ACP was established through the trifurcated graft by clamping its trunk and maintaining axillary artery flow. Next, a partial transverse incision was made either proximally in the old graft (Technique I) or in the aorta just distal to the old graft (Technique II, Video 1) depending on the length of ascending graft (Figure 2).^20^^,^^22^ Fenestration was created on the septum between true and false lumen. A classical elephant trunk (ET) was inserted into the descending thoracic aorta. This ET was anastomosed to the ascending graft and opening of the distal aorta or graft was closed using 3-0 polypropylene suture. Technique I required only single-layer closure (graft-to-graft) but Technique II required 2-layer of closure in the front (first layer, ET graft-to-old ascending graft, second layer, native aorta to native aorta). In patients who required proximal aortic operation or isolated arch pathology, aorta was transected at zone 2 or zone 3 level and standard distal anastomosis with insertion of classical ET was performed followed by ET to ascending graft anastomosis. Aortic crossclamp and antegrade cardioplegia were used in cases of concomitant aortic root procedure, coronary artery bypass grafting, or valve procedure.

#### Second-stage open repair

The second-stage repair was performed as a separate admission in most cases. Thoracoabdominal incision was made on sixth or seventh intercostal space. A spinal drain was placed in the operative room. The diaphragm was divided as necessary depending on distal extent of repair. Descending thoracic aorta was fully mobilized by sacrificing intercostal arteries from the outside. Neuromonitoring (motor- and sensory-evoked potential) was used and mean arterial pressure was maintained 85 to 90 mm Hg or greater to increase spinal cord perfusion. After systemic full heparinization, femorofemoral partial cardiopulmonary bypass (bladder temperature, 32 °C) was established with maintaining native cardiac output (approximately cardiac index of 1.0-1.2 L/min/m^2^). Previous ET was identified using epiaortic ultrasound. After reaching the target temperature, blood was drained from venous cannula which decreases mean arterial pressure to approximately 30 to 50 mm Hg temporarily. The descending aorta was longitudinally incised and the ET was manually grabbed and clamped. At this point, blood in a venous reservoir was returned to a patient to increase the arterial blood pressure. Distal aorta was prepared for anastomosis and then fenestration was created by removing the septal membrane. Open distal anastomosis was performed above the celiac axis using a Dacron graft. Lower body perfusion was resumed from femoral artery cannulation. Finally, proximal graft to ET anastomosis was performed (Video 2). The majority of repairs were extent I repair in this series.

### End Points and Definitions

The primary outcomes were operative mortality and aortic events. An aortic event was defined by unexplained sudden death, death related to aortic surgery or pathology, or any nonstaged aortic intervention. Planned stage II repair was not counted as an aortic event. Secondary outcomes were stroke, defined as new brain injury evident either clinically or radiographically, and spinal cord injury, defined as temporary or permanent paraplegia, or paraparesis. Computed tomography (CT) was performed before discharge or initial office visit. This was typically completed within a few weeks after the discharge as well as follow-up every several months. After 1 year of repair, follow-up was performed at the aortic clinic every 1 to 2 years, which included CT with an office visit or phone call follow-up. Four patients (5.3%) were lost to follow-up within 1 year after reoperation.

### Statistical Analysis

Continuous variables are presented as the median (first; third quartile) and were compared using the Mann-Whitney *U* test. Categorical variables are expressed in percentages and were compared using the χ^2^ or Fisher exact test (n < 5). The survival curve was analyzed by the Kaplan-Meier method with the log-rank test. Statistical analyses were performed using SPSS 24.0 (IBM Corp).

## Results

### Characteristics and Early Outcomes

The median age at operation was 63.5 years (Table 1). Male patients were predominant. Seven patients (11.3%) had connective tissue disorder. Five patients (8.9%) had multiple previous sternotomies before reoperative TAR. Six patients had history of endovascular repair for thoracic aorta (thoracic endovascular aneurysm repair [TEVAR]). Twenty-five patients (40.3%) had previous ATAD repair at outside hospital. The median interval from initial ATAD repair to reoperative TAR was 3 years [interquartile range, 1, 8] with maximal aortic arch diameter of 6.0 cm [5.5, 6.0]. Cardiac function was preserved in most patients.

**Table 1:** Demographics of patients

Variables	N = 62
Age, y	63.5 (53.8-71)
Male	44 (71%)
Body surface area, m^2^	2.0 (1.8, 2.2)
Body mass index, kg/m^2^	27.6 (24.4-32.6)
Connective tissue disease	7 (11.3%)
Smoking history	8 (16%)
Coronary artery disease	13 (21.0%)
Hypertension	56 (90.3%)
Diabetes mellitus	8 (12.9%)
Atrial fibrillation	9 (14.5%)
Chronic obstructive pulmonary disease	5 (8.1%)
Peripheral vascular disease	6 (9.7%)
Cerebrovascular disease	9 (14.5%)
Chronic kidney disease	5 (9.8%)
Ejection fraction, %	60 (55, 65)
Ejection fraction <50%	3 (4.8%)
Aortic insufficiency moderate or greater	8 (12.9%)
Maximal diameter of aorta, cm	6.0 (5.5-6.5)
Third sternotomy or more	5 (9.8%)
Interval from previous surgery, y	3.0 (1.0-8.0)
History of thoracic endovascular aortic repair	6 (9.7%)
Initial type A repair at outside hospital	25 (40.3%)
Maximal diameter at aortic arch, cm	6.0 (5.5, 6.0)

Values are expressed with n (%), or median (25%, 75% quartile).

### Operative Outcomes

The trifurcated graft and bifurcated graft technique were used in 51 patients (82.3%) and 11 patients (17.7%), respectively (Table 2). Concomitant procedure was performed in 20 patients (32.3%). Redo axillary cannulation was performed in 33 patients (53.2%). Single-patch reconstruction of left subclavian artery (LSCA) and left vertebral artery was performed in 2 patients.^22^

**Table 2:** Operative details of reoperative TAR

Characteristics	All, N = 62	Trifurcated graft group, n = 51	Bifurcated graft group, n = 11	*P* value
Concomitant procedure	20 (32.3%)	15 (29.4%)	5 (45.5%)	.499
Coronary artery bypass grafting	8 (12.9%)	8 (15.7%)	0	.362
Bentall procedure	7 (11.3%)	3 (5.9%)	4 (36.4%)	.018
Other procedure	6 (9.7%)	5 (9.8%)	1 (9.1%)	1.00
Redo axillary cannulation	33 (53.2%)	25 (49.0%)	8 (72.7%)	.153
Aberrant left vertebral artery	2 (3.2%)	2 (3.9%)	0	1.00
Minimum nasopharyngeal temperature, °C	16.9 (15.0-18.0)	17.0 (15.2-18.0)	16.7 (14.6-19.0)	.590
Minimum bladder temperature, °C	20 (19.0-21.3)	20.6 (19.2-21.8)	19.8 (18.7-20.0)	.026
SACP time, min	96 (74.8-103.3)	96 (75-104)	85 (61-101)	<.001
Lower body ischemic time, min	97 (83.5-103.3)	97 (85-104)	86 (63-101)	<.001
Cardiopulmonary bypass time, min	227.5 (204.8-258.8)	226 (205-249)	258 (202-302)	.285
Myocardial ischemic time, min	102 (92-143.8)	101 (93-139)	124 (63-200)	.507
pRBC, units	1 (0-3)	1 (0-3)	2 (0-3)	.567
Platelets, units	2 (2-4)	2 (0.25-4)	3 (2-4)	.293
Fresh-frozen plasma, units	0 (0-1)	0 (0-1.5)	0 (0-1.0)	.856
Cryoprecipitates, units	1 (0-3)	0.5 (0-2.75)	2 (0-3.5)	.665

Values are expressed with n (%), or median (25%, 75% quartile). *TAR*, Total arch repair; *SACP*, selective antegrade cerebral perfusion; *pRBC*, packed red blood cell.

The lowest nasopharyngeal and bladder temperatures were 16.9 °C and 20.0 °C, respectively. The cardiopulmonary bypass, myocardial ischemic, selective ACP, and lower body circulatory arrest times were 227.5, 102, 96, and 97 minutes, respectively. Selective ACP time (trifurcated, 96 [75-104] minutes vs bifurcated, 85 [61-101] minutes, *P* < .001) was shorter in the bifurcated graft group, as was lower body circulatory arrest time (97 [85-104] minutes vs 86 [63-101] minutes, *P* < .001). The bifurcated graft group had more concomitant Bentall procedure (5.9% [n = 3/51] vs 36.4% [n = 4/11], *P* = .018).

Operative mortality for reoperative TAR was 1.6% (n = 1/62), as was the incidence of stroke (1.6%, n = 1/62) and renal-replacement therapy (1.6%, n = 1/62). There was no spinal cord injury after reoperative TAR. Postoperative outcomes were comparable between the trifurcated and bifurcated graft group. Two patients required venoarterial extracorporeal membrane oxygenation support: 1 patient who had preoperative massive tricuspid regurgitation with reduced right ventricular function underwent tricuspid replacement, and the other with history of multiple sternotomies required complex proximal aortic root repair. Both patients were discharged and are continuing to do well. There was no right ventricular failure in isolated redo TAR patients.

Most recent CT angiography was available in 56 patients (median, 2.4 years [0.5-6.9 years] postrepair), and all anastomoses were patent in each group (Table 3). All head vessels were patent except for one case in the bifurcated group. This patient underwent carotid subclavian bypass and coiling of the LSCA at an outside hospital several years before reoperative TAR, and both the bypass and the LSCA were occluded.

**Table 3:** Hospital outcomes of reoperative TAR

Outcomes	All, N = 62	Trifurcated graft group, n = 51	Bifurcated graft group, n = 11	*P* value
Operative mortality	1 (1.6%)	1 (2.0%)	0	1.00
Stroke	1 (1.6%)	1 (2.0%)	0	1.00
Spinal cord ischemia	0	0	0	1.00
Reoperation for bleeding	3 (4.8%)	3 (5.9%)	0	.96
Tracheostomy	5 (8.1%)	4 (7.8%)	1 (9.1%)	1.00
Renal-replacement therapy	2 (3.2%)	2 (3.9%)	0	1.00
Bowel ischemia	0	0	0	1.00
Arm ischemia	0	0	0	1.00
Leg ischemia	0	0	0	1.00
Sternal wound complication	1 (1.6%)	1 (2.0%)	0	1.00
Brachial plexus symptom	1 (1.6%)	1 (2.0%)	0	1.00
Phrenic nerve injury	0	0	0	1.00
Neck swelling	1 (1.6%)	0	1 (9.1%)	1.00
Venoarterial extracorporeal membrane oxygenation	2 (3.2%)	1 (2.0%)	1 (9.1%)	1.00

	N = 56	Trifurcated graft group, n = 45	Bifurcated graft group, n = 11
Patency of neck vessels			
Innominate artery	56/56 (100%)	45/45 (100%)	11/11 (100%)
Left common carotid artery	56/56 (100%)	45/45 (100%)	11/11 (100%)
Left subclavian artery	55/56 (98.2%)	45/45 (100%)	10/11 (90.9%)
Patency of anastomosis			
Innominate artery	56/56 (100%)	45/45 (100%)	11/11 (100%)
Left common carotid artery	56/56 (100%)	45/45 (100%)	11/11 (100%)
Left subclavian artery	45/45 (100%)	45/45 (100%)	−

Values are expressed with n (%) or median (25%, 75% quartile). *TAR*, Total arch repair.

### Follow-Up outcomes

**Figure 3:**
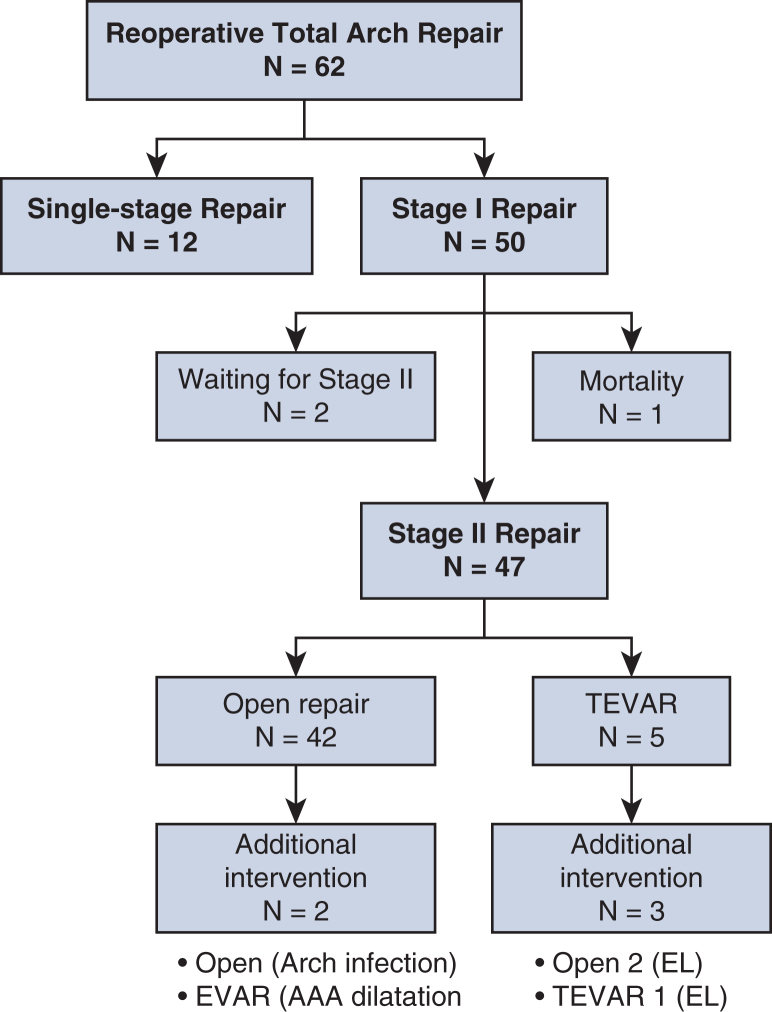
Flow chart of all patients. *TEVAR*, Thoracic endovascular aneurysm repair; *EL*, endoleak; *EVAR*, endovascular abdominal aortic repair.

There was no mortality while waiting for stage II repair (Table E1). Mean interval between staged procedures was 63 days [interquartile range, 36, 134]. Stage II repair was performed (n = 47: open repair, n = 42; endovascular repair, n = 3; and endovascular which eventually required open repair, n = 2) or planned (n = 2, waiting for open repair) in 49 patients (79.0%) (Figure 3). Mortality of stage II procedure was 4.3% (n = 2/47) with no spinal cord injury.

**Figure 4:**
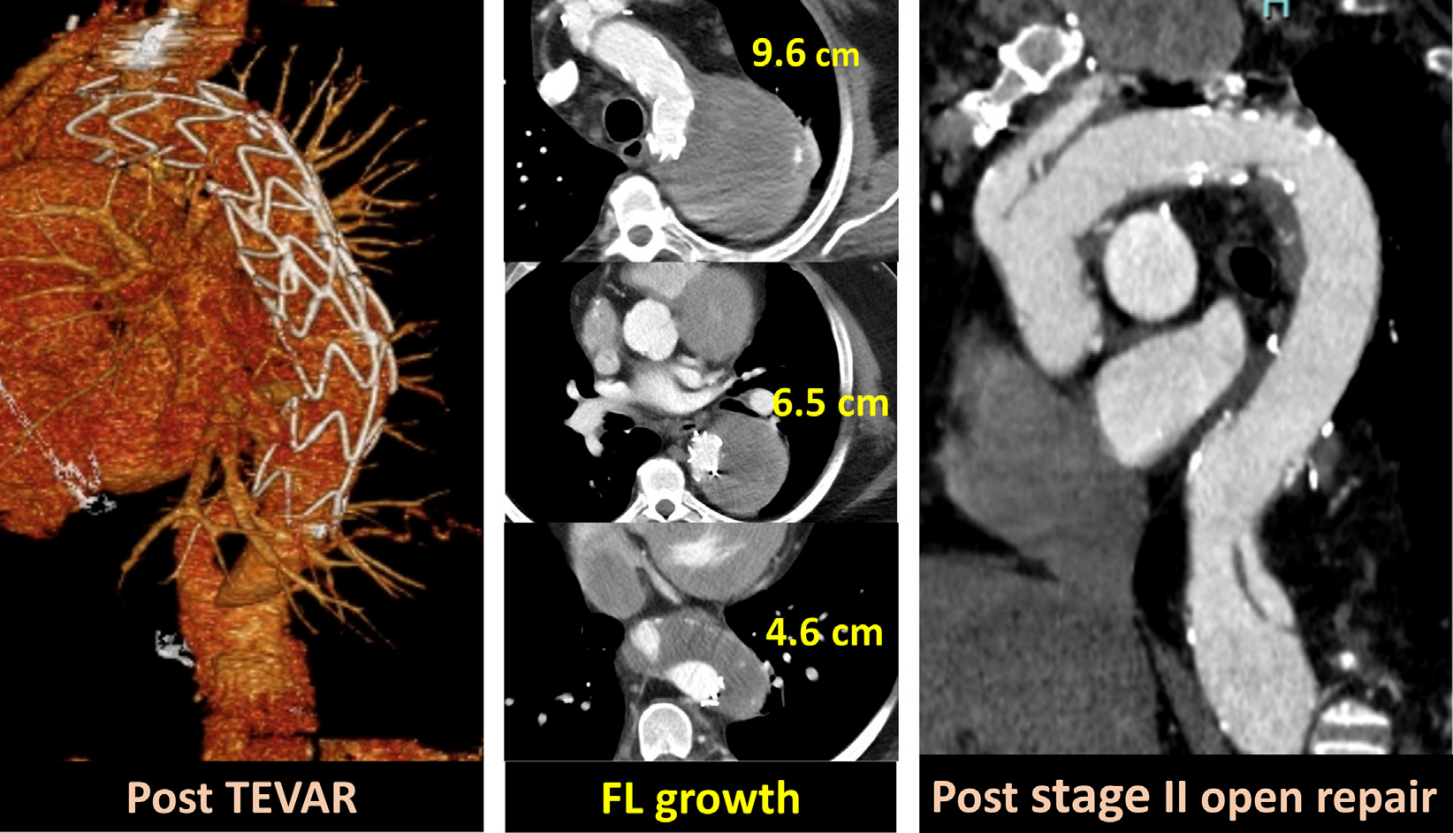
Computed tomography of a patient who underwent TEVAR. Open descending aortic repair was required due to expansion of downstream aorta 5 years after TEVAR. *TEVAR*, Thoracic endovascular aneurysm repair.

**Figure E1:**
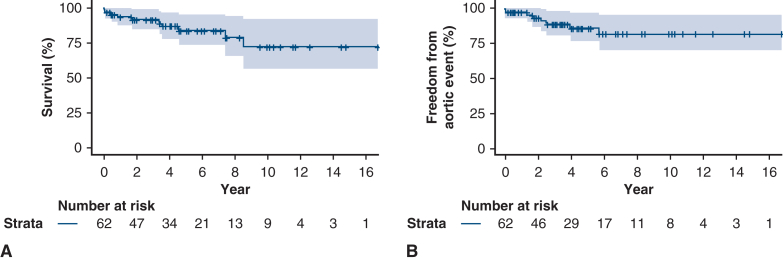
A, Kaplan-Meier survival curve showed that estimated survival at 3 and 5 years was 91.4 ± 3.7% and 83.7 ± 5.5%, respectively. B, Similarly, freedom from aortic reoperation was 88.7 ± 4.4% and 85.8 ± 5.1% at 3 and 5 years, respectively.

Median follow-up was 4.1 years. Kaplan-Meier analysis showed that estimated survival at 3 and 5 years was 91.4 ± 3.7% and 83.7 ± 5.5%, respectively (Figure E1, *A*). Freedom from aortic reoperation 3 and 5 years was 88.7 ± 4.4% and 85.8 ± 5.1%, respectively (Figure E1, *B*). After the survivors of staged II repair (n = 45), additional aortic operations were required in 5 patients: n = 2/40 after open repair, and n = 3/5 after TEVAR. The details of 2 aortic interventions after open repair as the stage II repair are open arch repair for infected ascending and arch graft (n = 1), and endovascular repair of abdominal aortic aneurysm as the result of dilatation (n = 1). Among 5 patients who had TEVAR as the stage II repair, 3 patients (60%) required additional aortic repair during follow-up, which includes open descending repair after TEVAR (n = 2) (Figure 4), and TEVAR after TEVAR for endoleak (n = 1).

## Discussion

There has been a perception that reoperative TAR after ATAD is a high-risk procedure. In the present study, we demonstrated the safety and efficacy of our reoperative TAR repair. The features of our strategy are: minimal dissection of proximal aorta (no need of aortic crossclamping in isolated reoperative TAR), reliable organ protection (ACP, deep hypothermia, and hyperkalemia followed by retrograde cardioplegia) and excellent surgical exposure (arch-first technique with lower body circulatory arrest).

The Cornell group showed favorable outcomes of reoperative TAR using deep hypothermia and retrograde cerebral perfusion.^11^ The benefits of their approach including minimal manipulation of head likely resulted in a low stroke rate. Despite the difference of cerebral perfusion strategy between their approach (retrograde cerebral perfusion) and ours (bilateral ACP), both favorable outcomes may support the safety and efficacy of deep hypothermia in this complex aortic repair where organ protection is the key component.

One of the benefits of using a trifurcated graft is versatility. The bifurcated graft technique was used in 17.7% of patients in the present series. This approach with preoperative carotid-LSCA bypass may be useful in patients with a posteriorly located LSCA, deep chest, or complex arch pathology such as concomitant proximal operation, multiple redo, infection, removal of TEVAR graft, or estimated long circulatory arrest time of the lower body.^23^ In our series, concomitant Bentall procedure was more predominant in the bifurcated group (36.4% vs 5.9%) compared with the trifurcated group. Both selective ACP and lower body circulatory arrest time were shorter in the bifurcated group due to one less head vessel anastomosis which would be useful in complex aortic repairs to minimize ischemic time. A potential downside of staged carotid-subclavian bypass is procedure-specific complications such as stroke, phrenic nerve injury, neck hematoma/lymphatic leak, occlusion of extra-anatomical bypass, or pseudoaneurysm.^23^ In the present series, 1 patient with a preoperative bypass procedure developed neck swelling from lymphatic leakage which resolved without any intervention. Since the Duke group reported that the incidence of the procedure-related complication after carotid-subclavian bypass was 29%, its indication needs to be discussed via case-by-case approach.^23^ Overall, patency rates of anastomoses are excellent regardless of a trifurcated or bifurcated technique which can be a potential benefit as determination of length of neck vessels in TAR using a four-branched arch graft may not be easy.^24^ Another example of the versatility is that a trifurcated graft can be flipped and a 12-mm limb can be used to reconstruct aberrant left vertebral artery and LSCA as a single patch, which was performed in 2 patients.^22^

One of the potential concerns of our techniques is ischemic time of the lower body, which persists until the completion of proximal anastomosis of the trifurcated graft.^9^ Etz and colleagues^25^ demonstrated that ACP with a rectal temperature of 28 °C provides the safe limit of 90 minutes as for spinal cord ischemia. In their logarithmic scale, 120 minutes at the 20 °C point falls within the safe time, which is the landmark of our safe limit of lower body ischemic time in reoperative TAR. In both trifurcated and bifurcated groups, lower body ischemic time was within this limit. In terms of other visceral organs, deep hypothermia was the only strategy for organ protection. The rate of renal failure (3.2%) were favorable. There was no liver failure in this series.

Although the majority of our patients underwent open repair as the second-stage procedure, there is growing interest in endovascular approaches, with advent of technology including the branched device, to treat complex pathology.^10^, ^11^, ^12^, ^13^, ^14^, ^15^ In our series, 3 of 5 patients who underwent TEVAR as a second stage repair required additional intervention including 2 open conversions. We consider that TEVAR is still challenging in chronic aortic dissection because of the following issues; complete coverage of entry tears, creation of optimal proximal and distal landing zone, preservation of head vessels and visceral branches, clot burden in the false lumen, multiple re-entries in the downstream aorta, and increased complexity of open repair if reoperation is necessary after TEVAR.^12^, ^13^, ^14^, ^15^^,^^17^

We prefer a 2-staged approach rather than extensive single-stage repair such as hemi-clamshell or clamshell incision because of a concern of early complications such as respiratory failure, bleeding, or wound complication.^17^^,^^25^ Although Kouchoukos and colleagues^26^ reported excellent long-term results of single-stage extensive repair, incidence of prolonged ventilation and tracheostomy were significant likely as the result of extensive exposure via bilateral thoracotomy.

Finally, the early outcomes of a hybrid bare stent (Ascyrus Medical Dissection Stent; Artivion) was recently reported, which forces the true lumen to the aortic wall with the potential distal aortic remodeling by sealing distal anastomosis.^27^ Long-term results regarding reintervention rate are warranted because this device also does not completely prevent new distal anastomotic entries, and redo operations after this device placement will be more challenging.^27^

Limitations of this study include that this is a single-center, retrospective study with a small number of patients without any reference group to compare differences of techniques such as different cerebral and spinal protection strategy (moderate hypothermia with direct cannulation of neck vessels, etc), distal repair using frozen ET, single-stage repair, hybrid, or total endovascular repair.

In conclusion, our strategy of reoperative TAR is safe and durable in the setting of residual dissection after ATAD repair. Reoperative TAR using a trifurcated graft enables in situ repair whereas a bifurcated graft technique allows a shorter circulatory arrest time of the lower body, which might be beneficial in patients who need concomitant complex proximal or arch repair. Insertion of classical ET followed by staged distal intervention showed favorable follow-up outcomes.

## Conflict of Interest Statement

The authors reported no conflicts of interest.

The *Journal* policy requires editors and reviewers to disclose conflicts of interest and to decline handling or reviewing manuscripts for which they may have a conflict of interest. The editors and reviewers of this article have no conflicts of interest.

## Figures and Tables

**Table E1:** Hospital outcomes of stage II repair

	n = 47
Open repair/TEVAR	42/5
Interval between stage I and stage II repair, d	63 [36-134]
Stage II repair during same admission of stage I repair	11 (29.7%)
Hospital mortality	2 (4.3%)
Stroke	1 (2.1%)
Spinal cord ischemia	0 (0%)
Tracheostomy	6 (12.8%)
Renal-replacement therapy	1 (2.1%)

*TEVAR*, Thoracic endovascular aortic repair.

## Supplementary Data

Video 1 Arch-first reoperative total arch repair. A partial transverse incision was made in the aorta just distal to the old graft (Technique II). Video available at: https://www.jtcvs.org/article/S2666-2507(25)00136-1/fulltext.

Video 2 Stage II open repair (extent I repair). Video available at: https://www.jtcvs.org/article/S2666-2507(25)00136-1/fulltext.
