# Supplementary figures and images for: Reoperative arch-first total arch repair after previous acute type A aortic dissection repair
Source: JTCVS Tech. 2025 Mar 28;31:1–10. doi: 10.1016/j.xjtc.2025.03.015 (PMC12237873; doi:10.1016/j.xjtc.2025.03.015)

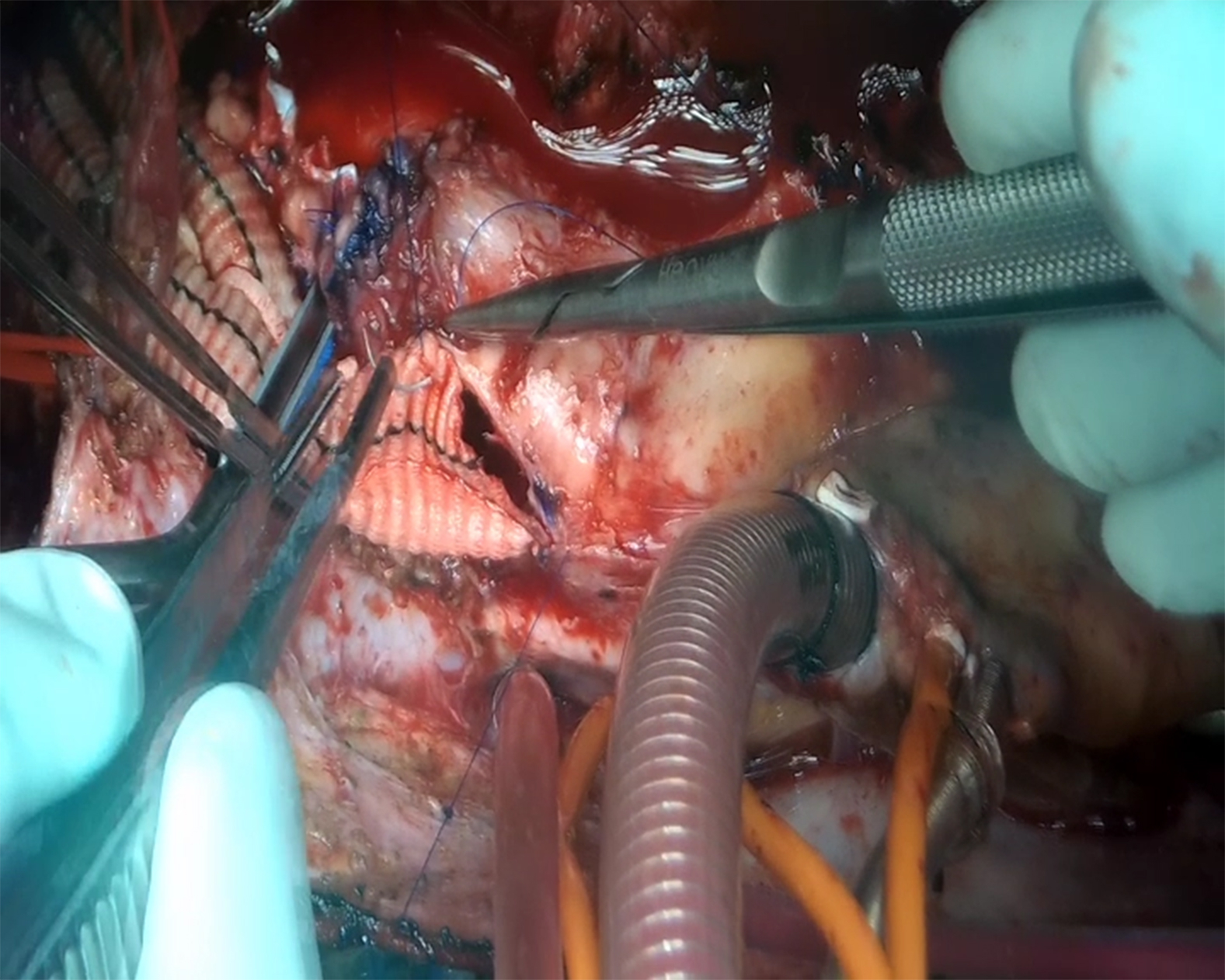

Supplement: Video 1 — Arch-first reoperative total arch repair. A partial transverse incision was made in the aorta just distal to the old graft (Technique II). Video available at: https://www.jtcvs.org/article/S2666-2507(25)00136-1/fulltext. [file fx2.jpg]

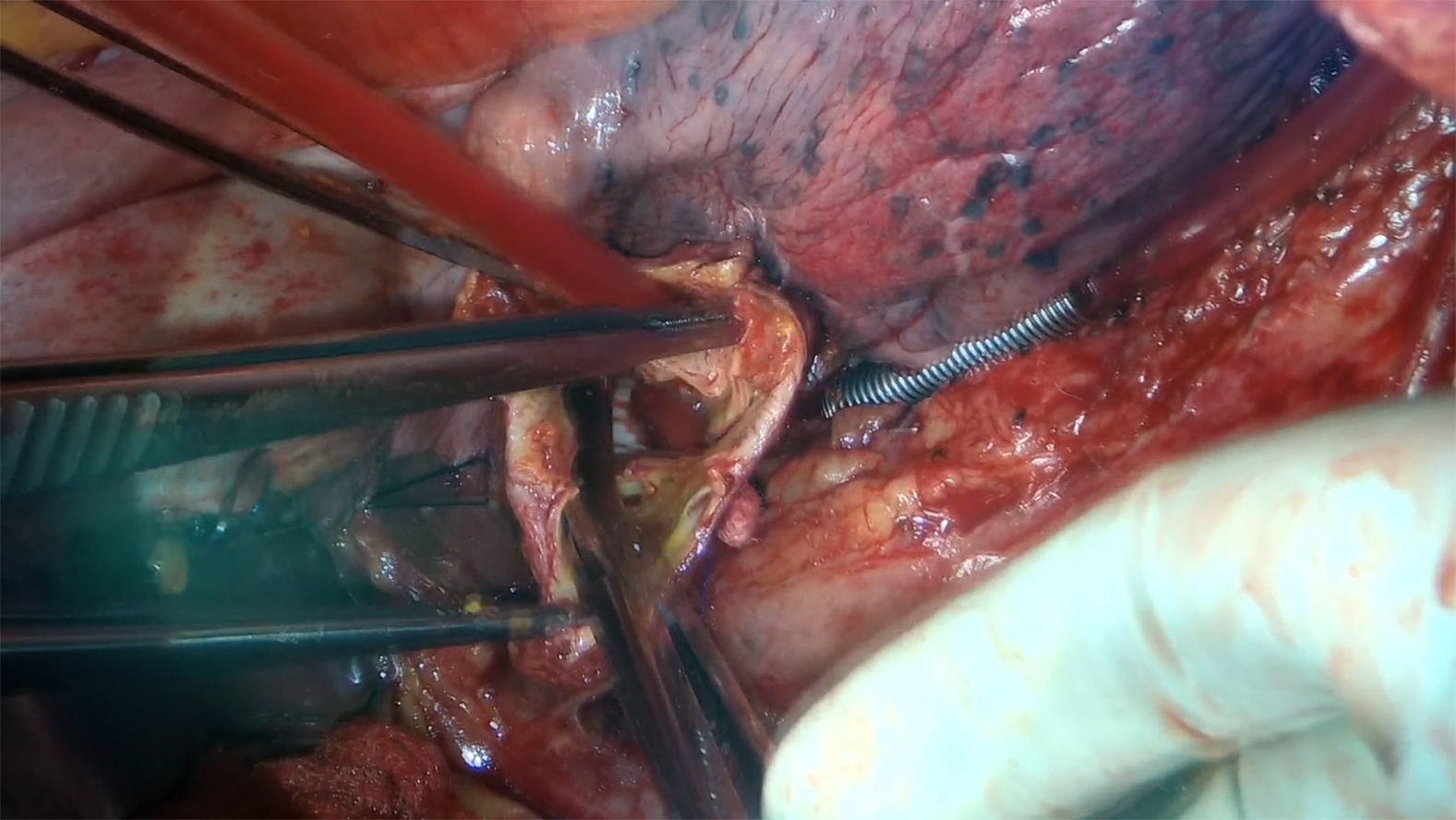

Supplement: Video 2 — Stage II open repair (extent I repair). Video available at: https://www.jtcvs.org/article/S2666-2507(25)00136-1/fulltext. [file fx3.jpg]
